# Gastrointestinal symptoms among recreational long distance runners in China: prevalence, severity, and contributing factors

**DOI:** 10.3389/fnut.2025.1589344

**Published:** 2025-07-23

**Authors:** Xueyuan Zhao, Yan Chen, Xiangxin Li, Wei Wen, Jingyi Zhang, Junqiang Qiu

**Affiliations:** ^1^School of Education, Beijing Sport University, Beijing, China; ^2^School of Sport Science, Beijing Sport University, Beijing, China; ^3^Beijing Sports Nutrition Engineering Research Center, Beijing, China

**Keywords:** marathon, running, gastrointestinal symptoms, nutrition strategies, prevalence

## Abstract

**Purpose:**

Gastrointestinal (GI) symptoms are prevalent among endurance athletes, especially marathon runners, and can negatively affect performance and wellbeing. However, data on the prevalence and nutritional contributors to GI symptoms in Chinese recreational long distance runners remain limited. This study aimed to investigate the prevalence, severity, and dietary influences of GI symptoms in this population.

**Methodology:**

A total of 805 valid responses were collected through an online and offline questionnaire conducted in China between January and December 2024. Participants were recreational long distance runners recruited via running clubs, community organizations, sports associations, and online platforms. The questionnaire covered six areas: demographics, exercise habits, dietary strategies, GI symptom severity and frequency, influencing factors, and knowledge and attitudes. GI symptoms during races were assessed using the Gastrointestinal Symptom Rating Scale (GSRS), which includes 11 symptoms rated on a 7-point Likert scale.

**Results:**

Notably, 26.1% of participants reported GI symptoms during races, with bloating (18.6%), urge to defecate (17.8%), and stomach pain (16.5%) being the most frequent. Symptoms peaked in prevalence and severity during the middle phase of the race. GI symptoms were more common in males (27.9%) than females (20.8%), and runners aged 34 years and younger had a higher symptom rates. Nutritional factors, particularly pre- and mid-race dietary strategies, significantly influenced symptom occurrence. Foods high in fat, protein, fiber, or fermentable carbohydrates were commonly associated with GI distress. Eating within 30 min before a race increased the risk of bloating and urge to defecate. The use of energy gels, sports drinks, and hydration strategies also correlated with higher GI symptom risk. Other contributing factors included a history of GI issues and high monthly running volume.

**Discussion:**

The findings underscore the importance of individualized dietary planning to reduce GI symptoms among recreational long distance runners. Adjusting pre-race meal timing and avoiding certain food types may mitigate discomfort. In addition to nutrition, variables such as sex, age, training load, and medical history should be considered in preventive strategies. Future research should explore tailored nutrition and training approaches to improve athlete health and performance during endurance events.

## 1 Introduction

Marathon running is widely recognized for its physical and mental health benefits, including improved cardiovascular fitness and reduced risk of chronic diseases. Prolonged and intense endurance exercise may also lead to adverse effects such as gastrointestinal (GI) symptoms (1, 2). Studies indicate that nearly half of marathon runners report experiencing occasional or frequent GI discomfort during or after running (3). These symptoms, which range from bloating and nausea to severe abdominal pain and diarrhea, present significant challenges for marathon participants (4, 5). Most studies primarily attribute these symptoms to the redistribution of blood flow to skeletal muscles during exercise, resulting in reduced splanchnic perfusion (1, 6, 7). This may contribute to increased mucosal permeability and promote the translocation of luminal endotoxins into the systemic circulation, potentially eliciting GI and/or systemic symptoms. (1, 8, 9). Additionally, factors such as exercise intensity and environmental temperature and humidity may exacerbate symptom severity (1, 10–12).

Considering the high energy demands of prolonged exercise, nutritional interventions, particularly dietary intake, and nutritional status, are especially important for long distance runners (13). However, the prevalence of GI symptoms is often the result of interactions among multiple nutritional factors (14). Dietary intake before and during the race, and even in the absence of food consumption, may influence GI symptoms during a marathon (4). It is currently known that carbohydrate (CHO) intake before and after endurance exercise can enhance performance but is also associated with GI symptoms, with potential mechanisms including malabsorption, delayed gastric emptying, among others (15). Differences in dietary patterns across countries may also have a potential impact on GI symptoms during exercise. For example, Western diets, which are rich in meat, high-fat foods, refined grains, and sugary beverages, are difficult to digest and may lead to delayed gastric emptying and GI discomfort (16, 17). In contrast, the Chinese diet is shaped by diverse geographical environments, multi-ethnic cultures, and historical traditions, resulting in unique dietary patterns that include a higher intake of whole grains, plant-based foods compared to Western diets (18, 19). However, it remains unknown whether the incidence of GI symptoms among Chinese runners is influenced by this context. In addition, recent studies have highlighted that a high intake of fermentable oligosaccharides, disaccharides, monosaccharides, and polyols (FODMAPs) may exacerbate GI symptoms, particularly in endurance athletes (20–22). Given that plant-based diets are common in the Chinese population, and plant-based diets are often rich in FODMAPs, their potential impact on GI symptoms in Chinese long distance runners warrants further investigation.

Despite extensive research on GI symptoms and their contributing factors among endurance runners (23, 24), no studies to date have specifically investigated these symptoms in Chinese runners. This gap highlights the need for further studies targeting this population to support the development of effective prevention and management strategies. Therefore, this study aims to investigate the prevalence, severity, and influencing factors of GI symptoms among Chinese recreational long distance runners, with a particular focus on examining the impact of nutritional factors on symptom occurrence and severity. It is hypothesized that certain specific foods, meal timing, and individual factors may be significantly associated with the occurrence and severity of GI symptoms among Chinese long distance runners.

## 2 Materials and methods

### 2.1 Participants

The China Marathon Nutrition Survey (CMNS) is a large-scale, cross-sectional project designed to investigate the nutritional status of Chinese long distance runners. The present study was developed as a sub-study within the CMNS framework and focuses specifically on GI symptoms and their nutritional correlates. The research protocol, approved by the Ethics Committee of Beijing Sport University (Approval Number: 2024256H), was conducted from January to December 2024. The participants of this study were long distance runners who had taken part in various endurance running events, including full marathons, half marathons, and other related races such as 10 km races and trail running events. Recruitment was conducted through three channels: running clubs, community organizations, sports associations, and the internet, ensuring a broad and representative sample. Inclusion criteria included participants aged 18 years or older, of any gender, who had participated in at least one full marathon, half marathon, or other types of marathon event, and were in good health without any serious diseases. Exclusion criteria included individuals with severe cardiovascular or cerebrovascular diseases, abnormal liver or kidney function, use of medications that may affect nutrition or metabolism (e.g., antibiotics, antidepressants, and antidiabetic drugs), as well as pregnant or breastfeeding women. These criteria were applied to minimize potential confounding effects from underlying health conditions or medications that might independently influence GI function. Pregnant and breastfeeding women were also excluded for ethical reasons and to reduce physiological variability unrelated to exercise or diet. To further ensure data consistency, individuals who had experienced significant weight changes within the past 3 months were also excluded. The surveyed population covered different genders, age groups, and levels of physical activity. Participants were divided into five groups based on their age at the time of the survey (18–34, 35–44, 45–54, 55–64, and ≥65 years). It should be noted that the age classification was determined by the participants’ age at the time of the survey, not their age at the time of their most recent marathon event. The training level was classified according to the Chinese Athletics Association Public Road Running Skill Level Standards (2023), categorizing recreational runners into Public Elite, Public Level 1 (L1), and Public Level 2 (L2) based on gender and age (ix 1) (Supplementary Material 1). All participants provided informed consent prior to participation. They were informed of the purpose of the study, how their data would be used, confidentiality protections, and the voluntary nature of their participation, in accordance with the approved research protocol.

### 2.2 Questionnaire

The questionnaire used in this study was specifically developed to assess GI symptoms and related factors among long distance runners, and was designed based on the aims of the CMNS project. It comprised six sections covering runners’ demographic information (gender, age, height, weight, and BMI), exercise habits (training level, years of running experience, mileage, number of races participated in, and common race types), dietary habits (pre-race meal timing, dietary restrictions before races, and food consumed during races), severity and frequency of GI symptoms during races, factors related to these symptoms (previous GI issues, pre-race sleep duration and quality, and pre-race anxiety levels), and knowledge and attitudes toward GI issues (causes and relief strategies) (ix 2) (Supplementary Material 2). The questionnaire was reviewed by experts and revised during the pre-survey phase to ensure scientific validity, logical clarity, and practical applicability.

GI symptoms during the race were evaluated using the Gastrointestinal Symptom Rating Scale (GSRS) (25, 26), a validated instrument that includes 11 common symptoms such as bloating, stomach pain, and urge to defecate. Each symptom was rated on a 7-point Likert scale, where 1 indicates no discomfort and 7 indicates severe discomfort. The GSRS has been demonstrated to reliably quantify GI distress and provides a standardized method to assess symptom prevalence and severity during endurance exercise.

### 2.3 Data collection and clearance

Data collection was carried out through a combination of online and offline methods. Generally, the sample size should be 5–10 times the number of items when developing a questionnaire (27). Based on the number of items in this questionnaire, a minimum of 320 responses was necessary. To ensure adequate coverage and representativeness, the questionnaire was widely distributed through both online and offline channels. Online distribution was conducted via official social media platforms, running club groups, and marathon registration platforms, where the questionnaire link was publicly and randomly distributed within these communities and completed voluntarily. Offline distribution was conducted on-site at selected marathon events and was limited to runners participating in those events. To ensure the authenticity and completeness of responses, online participants received detailed written instructions, while offline participants were given verbal guidance by trained personnel prior to and during questionnaire completion.

A total of 929 questionnaires were collected in this study. The data cleaning process strictly adhered to scientific standards and was conducted in two stages to ensure data accuracy and reliability. Initially, we excluded seven questionnaires that did not meet the inclusion criteria, such as participants under the age of 18. Subsequently, from the remaining questionnaires, we further eliminated those that were incomplete, inconsistent, or contained logical contradictions (*n* = 117). After rigorous screening, 805 valid questionnaires were ultimately included, achieving an effective rate of 86.7%. In the 805 questionnaires, Cronbach’s alpha was 0.788, the Kaiser–Meyer–Olkin (KMO) was 0.839, and Bartlett’s test yielded a significant result (χ^2^ = 3,193.256, df = 496, *p* < 0.001), confirming the questionnaire’s good reliability and validity.

### 2.4 Statistical analysis

The questionnaire data were statistically analyzed using Microsoft Excel 16.78 (Microsoft Corp., Redmond, WA, USA) and IBM SPSS Statistics 29.0 (IBM Corp., Armonk, NY, USA). Continuous variables, such as demographic characteristics, were described using mean ± standard deviation (mean ± SD), whereas categorical variables were expressed as frequency and percentage. Differences between groups based on sex, age, and exercise level were analyzed using the Chi-squared test (χ^2^) to assess the association between categorical variables. The independent samples *t*-test or Mann–Whitney *U* test was employed to examine differences between two groups, with group classification determined by the specific research question and variable of interest. Correlation analysis was performed using Pearson or Spearman rank correlation coefficients to evaluate the relationships between variables. Logistic regression analysis was used to explore the predictive role of dietary behavior on GI symptoms. All tests were two-tailed, and the significance level was set at *p* < 0.05.

## 3 Results

### 3.1 Participant demographics, anthropometrics, and training characteristics

A total of 805 valid questionnaires were analyzed. The majority of participants were male (74.9%, *n* = 603), with a mean age of 39.7 ± 10.0 years. Males were significantly younger than females (38.9 ± 10.2 vs. 41.9 ± 9.3 years, *p* < 0.001). They were also taller and heavier, resulting in a significantly higher BMI (23.0 ± 2.4 vs. 21.6 ± 3.1 kg/m^2^, *p* < 0.001). Most participants (82.1%) had a BMI in the normal range (18.5–24.9 kg/m^2^); however, 14.7% were overweight and 2.4% underweight. Overweight and obesity were more prevalent among men, while underweight was more common among women.

In terms of training and racing experience, 60.7% had less than 5 years of running experience, with the largest group (33.4%) reporting less than 3 years. Monthly running distance was most commonly in the 100–200 km range (43%). In terms of race participation, 40.4% had completed fewer than five marathons, and 28.0% had finished 6–10, indicating that most participants were still in the earlier stages of long-distance racing. Public L2 (28%) and L1 (25%) recreational runners made up the largest groups. Half-marathons were the most frequently attended event (64.6%), which may be attributed to their relatively greater accessibility and lower training requirements compared to full marathons. A full summary of participant demographics, anthropometrics, and running profiles is provided in Table 1.

**TABLE 1:** Demographic, measurement, and training-related information of runners.

Topic		Total 100% (805)	Male 74.9% (603)	Female 25.1% (202)
Height (cm)	Average	170.5 ± 7.5	173.4 ± 5.8	162.1 ± 5.1
Weight (kg)	Average	65.9 ± 10.0	69.1 ± 8.5	56.6 ± 8.1
Age (years)	Average	39.7 ± 10.0	38.9 ± 10.2	41.9 ± 9.3
	≤34	30.2% (243)	33.7% (203)	19.8% (40)
	35–44	37.4% (301)	36.2% (218)	41.1% (83)
	45–54	25.5% (205)	24.0% (145)	29.7% (60)
	55–64	6.5% (52)	5.6% (34)	8.9% (18)
	≥65	0.5% (4)	0.5% (3)	0.5% (1)
BMI (kg/m^2^)	Average	22.6 ± 4.3	23.0 ± 2.4	21.6 ± 3.1
Running experience (years)	≤3	33.4% (269)	33.2% (200)	34.2% (69)
	4–5	27.3% (220)	27.7% (167)	26.2% (53)
	6–7	15.7% (126)	15.8% (95)	15.3% (31)
	>7	23.6% (190)	23.4% (141)	24.3% (49)
Monthly running distance (km)	≤20	2.7% (22)	2.3% (14)	4.0% (8)
	20–50	7.3% (59)	6.3% (38)	10.4% (21)
	50–100	15.9% (128)	15.3% (92)	17.8% (36)
	100–200	43.0% (346)	43.4% (262)	41.6% (84)
	200–300	21.4% (172)	21.7% (131)	20.3% (41)
	300–500	7.8% (63)	8.6% (52)	5.4% (11)
	>500	1.9% (15)	2.3% (14)	0.5% (1)
Number of previous marathons	≤5	40.4% (325)	37.5% (226)	49.0% (99)
	6–10	28.0% (225)	30.5% (184)	20.3% (41)
	11–15	12.4% (100)	12.9% (78)	10.9% (22)
	16–20	7.6% (61)	7.5% (45)	7.9% (16)
	20–30	6.5% (52)	6.8% (41)	5.4% (11)
	>30	5.2% (42)	4.8% (29)	6.4% (13)
Training level	Public elite	23.1% (186)	24.9% (150)	17.8% (36)
	Public L1	25.0% (201)	24.7% (149)	25.7% (52)
	Public L2	28.0% (225)	29.9% (180)	22.3% (45)
	Unclassified	24.0% (193)	20.6% (124)	34.2% (69)
Types of frequent participation	Marathon	52.8% (425)	54.6% (329)	47.5% (96)
	Half marathon	64.6% (520)	65.0% (392)	63.4% (128)
	Others	9.3% (75)	6.2% (44)	11.8% (31)

Results are presented as percentages (%), total numbers, and means ± SD.

### 3.2 Prevalence, severity, and influencing factors of GI symptoms

Among all participants, 26.1% (*n* = 210) reported experiencing GI symptoms during races. The most common symptoms were bloating (18.6%), urge to defecate (17.8%), and stomach pain (16.5%) (Table 2). Symptom patterns varied by sex: males reported bloating (19.6%) and stomach pain (18.1%) as the most frequent upper GI symptoms, while urge to defecate (18.9%) and diarrhea (16.9%) were the most frequent lower GI issues. In females, bloating (15.8%) was also the leading upper GI complaint, followed by stomach pain and belching (both 11.9%). For lower GI symptoms, urge to defecate (14.4%) and abdominal pain or side stitch (12.4%) were more common than diarrhea.

**TABLE 2:** Prevalence of symptom scores: mild (≥2), moderate (≥4), and severe (≥6) symptoms (*n* = 805).

Symptom	Total	Mild (≥2)	Moderate (≥4)	Severe (≥6)
Heartburn	15.0% (121)	10.4% (84)	4.2% (34)	0.4% (3)
Belching	15.5% (125)	11.2% (90)	4.0% (32)	0.4% (3)
Bloating	18.6% (150)	13.3% (107)	4.8% (39)	0.5% (4)
Stomach pain	16.5% (133)	11.9% (96)	4.0% (32)	0.6% (5)
Nausea	13.8% (111)	10.4% (84)	3.0% (24)	0.4% (3)
Vomiting	13.8% (111)	10.9% (88)	2.7% (22)	0.1% (1)
Intestinal spasm	12.8% (103)	9.9% (80)	2.7% (22)	0.1% (1)
Urge to defecate	17.8% (143)	10.9% (88)	6.3% (51)	0.5% (4)
Abdominal pain/side stitch	15.3% (123)	10.3% (83)	4.3% (35)	0.6% (5)
Flatulence	10.8% (87)	7.5% (60)	3.0% (24)	0.4% (3)
Diarrhea	15.4% (124)	11.1% (89)	4.2% (34)	0.1% (1)

**FIGURE 1:**
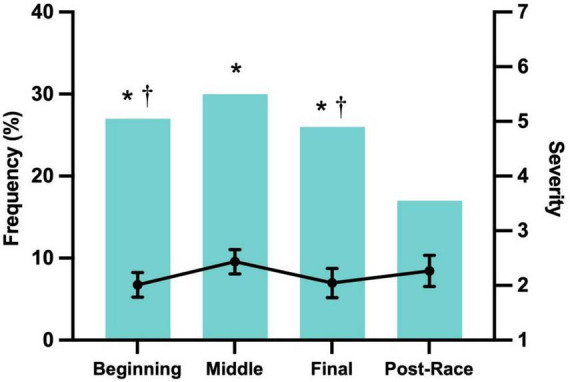
Frequency and severity on a scale of 0 (“none”) to 1 (“very severe”) by segment for finishers who reported that GI symptoms affected race performance. Severity data represent mean scores calculated only from participants who reported symptoms of at least mild intensity in any race segment. *Frequency was significantly greater than the Post-Race Stage (*p* < 0.05). ^†^ Frequency significantly greater than that in the Middle Stage (*p* < 0.05).

Most symptoms were mild in severity (score ≥ 2), with bloating being the most frequently reported mild symptom (13.3%). Moderate symptoms (score ≥ 4) were less common, led by urge to defecate (6.3%), while severe symptoms (score ≥ 6) were rare, occurring in less than 1% of participants, and primarily involved stomach or abdominal pain. The frequency and severity of GI symptoms varied across different race stages (Figure 1). Symptoms peaked during the middle stage (30.0%), which also had the highest severity score (2.43 ± 0.22), and were least frequent after the race (16.7%), though post-race symptoms showed relatively high severity (2.26 ± 0.29). Female participants reported a higher incidence of symptoms in the initial stage (33.3%) than males (25.6%), while runners with unclassified training levels had the highest symptom frequency in the final stage (38.6%).

Several factors were significantly associated with the occurrence of GI symptoms (Table 3). Males showed a higher prevalence than females (27.9% vs. 20.8%, *p* = 0.048), and runners aged 34 or younger were more likely to report symptoms (*p* = 0.014). A self-reported history of GI disorders—including clinically diagnosed or perceived conditions such as gastritis, functional dyspepsia, irritable bowel syndrome, or chronic constipation—was the strongest predictor (*p* < 0.001). Monthly running volume was also associated with symptom prevalence, with runners averaging 20–50 km or more than 500 km per month showing increased risk (*p* = 0.015). No significant associations were observed with running experience, training level, race history, or pre-race sleep and stress levels.

**TABLE 3:** Impact of related factors on GI symptoms during race.

Related factors	GI symptoms during race		
	χ^2^	df	*p*
Gender	3.921	1	0.048
Age	12.574	4	0.014
Running experience	0.104	3	0.991
Monthly running distance	15.856	6	0.015
Training level	3.911	3	0.271
Number of previous marathons	5.869	5	0.319
Prior GI symptoms	87.468	1	<0.001
Pre-race sleep duration and quality	2.386	5	0.794
Pre-race stress level	5.074	3	0.166

### 3.3 The impact of nutritional strategies on GI symptoms

**FIGURE 2:**
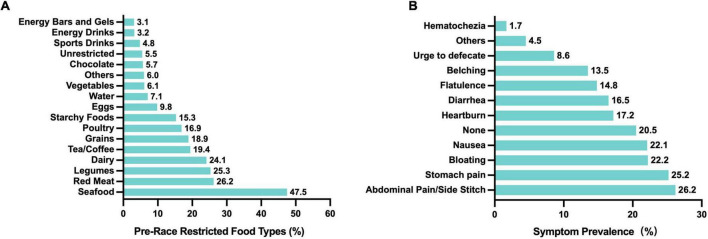
Food restrictions before races and associated GI symptoms in Chinese recreational long distance runners. **(A)** Types of foods typically restricted before races (*n* = 805). **(B)** Symptoms during the race without pre-race food restrictions (*n* = 761).

To minimize the risk of GI discomfort during races, most runners reported restricting certain foods beforehand. Among 805 participants, only 5.5% reported no dietary restrictions (Figure 2A). The most commonly avoided items were seafood (47.5%), red meat (26.2%), legumes (25.3%), dairy (24.1%), and tea/coffee (19.4%), while avoidance rates for energy bars/gels (3.1%), energy drinks (3.2%), and sports drinks (4.8%) were much lower. Differences by sex in food avoidance are detailed in Table 4. These patterns suggest that runners aim to avoid foods that are harder to digest or more likely to cause GI upset. Nevertheless, among runners who did not restrict their diet before the race, common symptoms included abdominal pain or side stitches (26.2%), stomach pain (25.2%), bloating (22.2%), and nausea (22.1%) (Figure 2B). Severe symptoms such as hematochezia were rare (1.7%), and 20.5% of runners reported no symptoms despite not restricting their diet.

**TABLE 4:** Types of foods restricted before races by male and female Chinese recreational long distance runners.

Foods	Male (*n* = 603)	Female (*n* = 202)
Seafood	47.9% (289)	46.0% (93)
Red meat	28.0% (169)	20.8% (42)
Legumes	23.9% (144)	29.7% (60)
Grains	19.2% (116)	17.8% (36)
Dairy	22.7% (137)	28.2% (57)
Starchy foods	15.8% (95)	13.9% (28)
Vegetables	6.5% (39)	5.0% (10)
Poultry	17.7% (107)	14.4% (29)
Eggs	10.8% (65)	6.9% (14)
Water	7.6% (46)	5.4% (11)
Energy bars and gels	3.3% (20)	2.5% (5)
Sports drinks	5.3% (32)	3.5% (7)
Energy drinks	3.8% (23)	1.5% (3)
Tea/coffee	19.4% (117)	19.3% (39)
Chocolate	6.8% (41)	2.5% (5)
Others	5.6% (34)	6.9% (14)
Unrestricted	5.0% (30)	6.9% (14)

Pre-race meal timing also influenced GI symptom occurrence. Eating within 30 min of race start was significantly associated with increased bloating (*p* = 0.017), urge to defecate (*p* = 0.040), and flatulence (*p* = 0.011) (χ^2^ = 21.660, df = 6, *p* = 0.001). Additionally, runners who experienced GI symptoms during the race were more likely to report appetite loss afterward (χ^2^ = 37.089, df = 3, *p* < 0.001), emphasizing the importance of careful meal timing for both performance and recovery.

**FIGURE 3:**
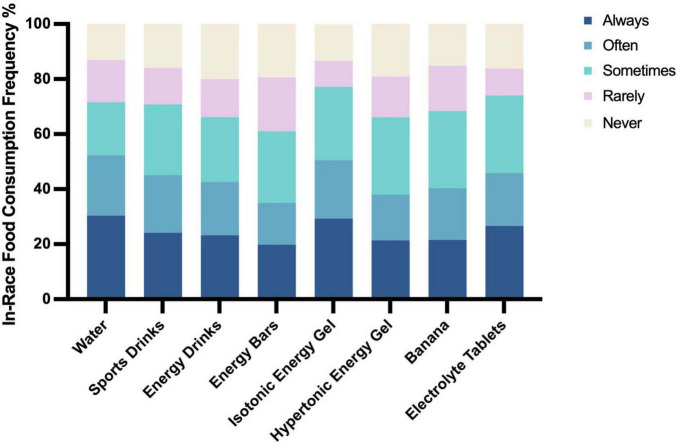
Types and frequency of food consumption during races in Chinese recreational long distance runners (*n* = 805).

During races, nutritional strategies varied widely. The most frequently consumed items were water (30.4%), isotonic energy gels (29.3%), sports drinks (26.6%), electrolyte tablets (24.1%), bananas (21.5%), and energy bars (26.0%) (Figure 3). Isotonic energy gels were more commonly used than hypertonic ones (29.3% vs. 21.4%). Sports drinks, which contain electrolytes and carbohydrates (24.1%), and energy drinks, which include caffeine, sugar, and stimulants (23.2%), were also popular, though the latter were used less consistently. Notably, consumption of water (*p* = 0.045), sports drinks (*p* = 0.012), energy drinks (*p* = 0.004), energy bars (*p* = 0.042), and isotonic gels (*p* = 0.002) was significantly associated with bloating during the race, suggesting that while these products support hydration and energy, they may also contribute to GI discomfort.

### 3.4 Runners’ awareness and management of GI symptoms

Among the 210 runners who reported GI symptoms, 76.2% indicated that symptoms negatively impacted their performance. The most common relief strategies included slowing down (76.2%) and walking or stopping for a break (41.0%), with 53.8% finding these measures effective. Additionally, 3% of symptomatic participants reported using non-steroidal anti-inflammatory drugs (NSAIDs) to manage symptoms, despite research suggesting potential risks to gut health (28, 29). Regarding sources of information about GI symptoms, among all 805 participants, 42.2% reported relying on social media, followed by books and magazines (37.8%) and conversations with friends and family (31.8%).

## 4 Discussion

To the best of our knowledge, this is the first and larges-scale study examining the prevalence and severity of GI symptoms among recreational long distance runners in China. Our results indicate that approximately 26% of runners experience GI symptoms, with bloating, stomach pain, and the urge to defecate being the most common. We identified that pre-race meal timing and food choices significantly influence the occurrence of these symptoms. Additionally, we also observed a U-shaped relationship between training volume and GI distress, with both low and very high training volumes associated with an increased likelihood of symptoms. Individual factors such as younger age and a history of GI issues were also found to be linked to a higher prevalence of these symptoms. These findings confirm our hypothesis regarding the contributing factors. Notably, although the Chinese diet is typically rich in whole grains and plant-based foods—both high in FODMAPs—the overall prevalence of GI symptoms in this population was not particularly elevated.

In line with previous observations, upper GI symptoms are typically more prevalent than lower GI symptoms during prolonged endurance exercise (24). However, the prevalence and severity of GI symptoms reported in different observational studies vary significantly, ranging from 4% to 96% (24, 30–32); duration, type of exercise, and environmental temperature are the main factors contributing to these variations (24). In ultramarathons and multi-stage races, the prevalence of symptoms can be as high as 60%–96% (31–33). The repetitive jumping motion in running is more likely to trigger symptoms than cycling (6, 34), and GI symptoms are more frequent and severe in hot environments compared to cold to thermally neutral environments (31). Additionally, related studies suggest that feeding during exercise, at a time when the GI tract is compromised, may act as a risk factor for the development or exacerbation of symptoms (24).

Building upon these findings, our study identified that nutritional factors, including dietary strategies before and during the race, are key contributors to GI symptoms among Chinese runners. High-FODMAP foods, which contain short-chain carbohydrates and are often restricted due to individual tolerance differences, are not fully absorbed in the small intestine and undergo fermentation in the colon, leading to gas production and GI discomfort (22, 35). Second, pre-race meal timing was also significantly associated with GI symptoms: eating within 30 min before the race was more likely to trigger bloating, an increased urge to defecate, and flatulence. The American College of Sports Medicine (ACSM) recommends consuming a full meal 3–4 h before exercise, while smaller meals or snacks should be consumed 2–4 h prior (36). Therefore, the rational arrangement of pre-race nutrition is a crucial strategy for reducing GI symptoms and enhancing competitive performance.

Certain supplements and fluids may also contribute to the development of GI symptoms. Our study found that water, sports/energy drinks, energy bars, and isotonic energy gels were more likely to cause bloating, which aligns with previous research (15). The mechanisms underlying exercise-induced GI symptoms are multifaceted, involving not only the type and quantity of CHO intake, but also factors such as the pH level of beverages, which may contribute to GI distress (14). Given these complexities, several nutritional strategies have been proposed for mitigating GI discomfort during exercise. “Training the Gut” is a strategy that involves gradually adapting the GI system to fluid and food intake, thereby improving tolerance, and reducing GI discomfort during races (5, 37). Additionally, a low-FODMAP diet is also a crucial approach to minimize GI symptoms (38, 39). It is noteworthy that certain nutritional supplements have shown potential benefits in some studies, though their long-term efficacy and safety still require further validation (1).

A notable finding in our study was the significant association between running volume and GI symptoms. Interestingly, we identified a U-shaped relationship, where both low (20–50 km per month) and very high (>500 km per month) training volumes were linked to a higher likelihood of experiencing GI symptoms. This suggests that both insufficient and excessive training volume may contribute to GI distress during races. Nevertheless, further research is needed to clarify the relationship between training-related factors and GI symptoms. In contrast, training level and running experience did not show a statistically significant correlation with GI symptoms. Public Elite runners had the highest reported incidence of symptoms (31.2%), but this difference was not statistically significant. This may be because factors such as training volume have a more direct impact on GI symptoms than training level or experience alone. Future studies are warranted to further explore the optimal range of training volume that minimizes GI distress while maintaining performance benefits.

Furthermore, individual factors also contribute to the prevalence and severity of GI symptoms. Younger age and history of GI issues are recognized risk factors for GI symptoms in endurance athletes, which is consistent with our study (15, 23, 40). However, contrary to prior research, we observe that female runners reported a lower GI symptom prevalence (20.8%) than male runners (27.9%). This discrepancy may stem from differences in sex ratios, regional populations, or training habits. A research indicate that female participation varies significantly in marathon, ranging from 16% to 21% in some Asian and European countries (e.g., Japan, South Korea, Switzerland, and Italy) to 52%–59% in Iceland, the United States, Canada, Ireland, and Australia (41). In our study, the proportion of female runners in China increased from 19.56% in 2019 to 25.1% in 2023 (42). As the number of women participating in the sport continues to grow, future research should focus on the GI issues faced by female runners to better understand the gender-specific dynamics of GI symptoms.

Chinese recreational runners in this study demonstrated a moderate awareness of exercise-induced GI issues. Among symptomatic runners, most reported a negative impact on performance and commonly used strategies like slowing down or taking breaks, with about half finding these effective. Notably, 3% used NSAIDs despite potential risks to gut health. Runners mainly obtained information from social media, books, and peers; however, social media content often lacks scientific rigor and can be misleading. This highlights the need for improved education on safe and effective management of GI symptoms. Professional organizations, sports science teams, and sports nutritionists should play a key role in raising awareness and helping runners better manage GI issues and reduce associated risks.

This study, as the first large-scale investigation of GI symptoms among Chinese recreational long distance runners, provides practical insights for personalized nutrition and training strategies. While the use of a validated questionnaire and diverse sample strengthens the findings, limitations include its cross-sectional design and reliance on self-reported data, which may introduce recall bias. The results highlight key factors such as pre-race meal timing, food choices, and training volume. Given the unique dietary patterns of Chinese runners—characterized by higher intake of plant-based and FODMAP-rich foods—the findings may not fully generalize to runners from other regions. Future research should explore cross-cultural comparisons and investigate physiological mechanisms to inform more targeted interventions.

## 5 Conclusion

This study found that GI symptoms are relatively common among Chinese recreational long distance runners, with bloating, urge to defecate, and stomach pain being the most frequently reported symptoms. Symptoms were most pronounced during the middle- and post-race stages, with nutritional factors playing a key role. A shorter pre-race meal interval and certain in-race nutritional choices were associated with increased symptoms. Additionally, other factors such as prior GI issues and training volume also influenced symptom occurrence. Future research should integrate additional biomarkers to examine and compare the prevalence of GI symptoms among runners across different regions, while investigating the mechanisms by which specific factors influence these symptoms. Furthermore, personalized intervention and prevention strategies should be developed to optimize the alleviation of GI discomfort, enhancing athletic performance and overall health.

## Data Availability

The original contributions presented in this study are included in this article/Supplementary material, further inquiries can be directed to the corresponding author.
