# Supplementary material for: Gastrointestinal symptoms among recreational long distance runners in China: prevalence, severity, and contributing factors
Source: Front Nutr. 2025 Jul 23;12:1589344. doi: 10.3389/fnut.2025.1589344 (PMC12325075; doi:10.3389/fnut.2025.1589344)
Supplement: Supplementary file 2 [file Table_2.docx]

**Gastrointestinal Symptoms and Nutrition Practices Questionnaire for Chinese Recreational Long Distance Runners**

This questionnaire aims to collect data on gastrointestinal symptoms and nutrition-related practices among recreational marathon runners in China. The study specifically targets this population to explore the prevalence, potential triggers, and self-management strategies related to gastrointestinal issues during endurance events. Findings from this research will offer valuable insights to sports scientists and healthcare professionals, enabling them to develop more tailored and culturally appropriate strategies to enhance gastrointestinal comfort and athletic performance in Chinese recreational runners.

All responses will be kept strictly confidential, and only members of the research team will have access to the data. Survey responses will be stored securely and used exclusively for academic and scientific purposes. Any reports or publications resulting from this study will ensure that individual participants cannot be identified.

Your participation in this survey is entirely voluntary, and you may withdraw at any time without penalty. Please ensure that you have signed the informed consent form before completing the questionnaire. If you decide to withdraw later, please use your participant number from the consent form.

**We greatly appreciate your honesty and cooperation in answering all questions as accurately as possible. Thank you for your participation.**

1. Please indicate your gender:

- Female
- Male

2. What is your current age (in years)? (Please write a number)__________

3. What is your current height (in centimeters)? (Please write a number) __________

4. What is your current weight (in kilograms)？(Please write a number) __________

5. What is your current training level, as classified by the Chinese Athletics Association Public Road Running Skill Level Standards (2023)?

- Public Elite
- Public Level 1
- Public Level 2
- None

6. How many years have you incorporated running into your regular training or exercise routine?

- 0 - 3 years
- 3 - 5 years
- 5 - 7 years
- More than 7 years

7. What has been your average monthly running mileage over the past three months (in kilometers)?

- less than 20 km
- 20 - 50 km
- 50 - 100 km
- 100 - 200 km
- 200 - 300 km
- 300 - 500 km
- More than 500 km

8. How many marathon events have you participated in?

- 0 - 5 races
- 6 - 10 races
- 11 - 15 races
- 16 - 20 races
- 20 - 30 races
- More than 30 races

9. Which type of marathon race do you participate in most often?

- marathon (42 km)
- ½ marathon (21 km)
- Ultra-Distance (> 42 km)
- Trail marathon (off-road)
- Road race (less than half marathon)
- Other (please specify): __________

10. Do you intentionally avoid any specific foods as part of your daily dietary habits?

- Yes
- No - Please skip to Question 11

10a. If yes, what are the reasons for avoiding these foods? (Select all that apply)

- Food allergy or intolerance
- History of adverse reactions or discomfort after consumption
- Personal dietary preference
- Recommendations from others (e.g., coach, healthcare provider, peers)
- Religious or cultural beliefs
- Other (please specify): _________

11. How long before the race do you usually consume your pre-race meal?

- Less than 30 minutes
- 30 minutes to 1 hour
- 1 - 2 hours
- 2 - 3 hours
- 3 - 4 hours
- More than 4 hours
- I do not eat anything on race morning (remain fasted) - Please skip to Question 12

11a. Which foods or beverages do you usually avoid in your pre-race meal? (Select all that apply):

- Seafood
- Red meat
- Legumes
- Grains
- Dairy products
- Starchy foods
- Vegetables
- Poultry
- Eggs
- Water
- Energy bars and gels
- Sports drinks
- Energy drinks
- Tea or coffee
- Chocolate
- Others (please specify): ________________
- I do not restrict any food or beverage

11b. If you consume any of the above foods or beverages, which of the following symptoms do you typically experience during a race? (Select all that apply):

- Heartburn
- Belching
- Bloating
- Stomach pain
- Abdominal pain / side stitch
- Nausea
- Diarrhea
- Flatulence
- Urge to defecate
- Hematochezia (blood in stool)
- None
- Others (please specify): ________________

12. Do you consume any of the following foods or beverages during the race?

|  | Never | Rarely | Sometimes | Often | Always |
| --- | --- | --- | --- | --- | --- |
| Water |  |  |  |  |  |
| Sports Drinks |  |  |  |  |  |
| Energy Drinks |  |  |  |  |  |
| Energy Bars |  |  |  |  |  |
| Isotonic Energy Gel |  |  |  |  |  |
| Hypertonic Energy Gel |  |  |  |  |  |
| Banana |  |  |  |  |  |
| Electrolyte Tablets |  |  |  |  |  |

13. How would you rate your sleep duration and quality the night before the race?

- More than 7 hours, good quality
- More than 7 hours, poor quality
- 5 to 7 hours, good quality
- 5 to 7 hours, poor quality
- Less than 5 hours, good quality
- Less than 5 hours, poor quality

14. How would you describe your level of nervousness before the race?

- Not nervous at all
- Slightly nervous
- Moderately nervous
- Very nervous

15. Have you previously experienced any gastrointestinal symptoms or disorders (e.g., gastritis, indigestion, irritable bowel syndrome, gastroesophageal reflux disease)?

- Yes
- No

16. How severe were the following gastrointestinal symptoms during the race?

|  | None | Very mild | Mild | Moderate | Moderate Severe | Severe | Very severe |
| --- | --- | --- | --- | --- | --- | --- | --- |
| Heartburn |  |  |  |  |  |  |  |
| Belching |  |  |  |  |  |  |  |
| Bloating |  |  |  |  |  |  |  |
| Stomach pain |  |  |  |  |  |  |  |
| Nausea |  |  |  |  |  |  |  |
| Vomiting |  |  |  |  |  |  |  |
| intestinal spasm |  |  |  |  |  |  |  |
| Urge to defecate |  |  |  |  |  |  |  |
| Abdominal Pain/Side Stitch |  |  |  |  |  |  |  |
| Flatulence |  |  |  |  |  |  |  |
| Diarrhea |  |  |  |  |  |  |  |

17. During which stage of the race do you experience gastrointestinal symptoms most frequently?

- Beginning
- Middle
- Final
- Post-race

18. Does gastrointestinal discomfort affect your race performance (e.g., causing interruptions or slowing down)?

- Never
- Sometimes
- Often
- Always

19. What strategies have you used to relieve gastrointestinal symptoms during running? (Select all that apply)

- Slowing down your running pace
- Walking or stopping to rest
- Adjusting your breathing during running
- Drinking water or sports drinks slowly
- Eating small amounts of food slowly
- Taking non-steroidal anti-inflammatory drugs (NSAIDs)
- No action taken
- Other (please specify): ________________

20. Through which sources have you obtained knowledge about gastrointestinal health? (Select all that apply)

- Family and friends
- Books or magazines
- Social media
- Coach or professional fitness instructor
- Sports nutritionist
- Dietitian or dietary expert
- Other (please specify): ________________
- None
